# Eph receptors as oncotargets

**DOI:** 10.18632/oncotarget.21045

**Published:** 2017-09-19

**Authors:** Sara Charmsaz, Andrew W. Boyd

**Affiliations:** Sara Charmsaz: Leukaemia Foundation of Queensland Laboratory, QIMR Berghofer Medical Research Institute, Brisbane, QLD, Australia; Department of Medicine, University of Queensland, Brisbane, QLD, Australia; Department of Surgery, Royal College of Surgeons, Dublin, Ireland

**Keywords:** Eph, EphA2, EphA3

The discovery of EphA1, a novel cancer-associated receptor tyrosine kinase (RTK), in 1987 paved the way to identification of the 16 members of the Eph RTK family. Many of these were initially identified in a variety of human cancers. Eph RTKs mainly function, during development, to regulate cell positioning and migration. However, Eph receptors and their ephrin ligands are also widely and aberrantly expressed in a number of diseases, in particular, in many human malignancies, where their level of expression often correlates with increased aggressiveness [1].

Eph family of RTKs have been shown to have a complex role in tumour formation, progression and metastasis. One aspect of this complexity is the potential for more than one of a number of Eph RTKs to be expressed in a particular tumour whilst exerting the same functional effect. A further aspect is that an Eph RTK can have either tumour promoting or inhibitory effects depending on the tumour type and the stage of the disease. The higher level of expression of the Eph receptors, compared with normal tissues, together with their functional role in cancer has led to an increased interest in developing therapeutic strategies targeting Eph proteins in many cancers [2].

Therapies targeting Eph receptors may operate through a number of mechanisms. Where Eph proteins function as tumour promoters, therapies that target these proteins by blocking their function or their downstream signalling mechanisms may be effective. Despite most Eph proteins being functional tyrosine kinases it has been demonstrated that oncogenic functions are largely kinase independent and hence kinase inhibitors are generally ineffective or even contraindicated [3-5]. Another approach has been to target these receptors with specific monoclonal antibodies either to inhibit function or to target the immune system or a toxic payload specifically to the tumour cells [4]. EphA2, EphA3 and EphB4 are some of the most widely over-expressed Eph RTKs in cancer. In many cases the expression level correlates with the aggressiveness of the tumour. Thus, these three Eph RTKs have been studied extensively as targeted cancer therapeutics.

EphA2 expression has been demonstrated in many cancers; including colon, skin, kidney, breast, glioblastoma and lung. Functionally, EphA2 expression has been shown to influence tumour angiogenesis, migration, proliferation and adhesion. Many strategies have been used for targeting EphA2, in both pre-clinical and clinical setting including the use of monoclonal antibodies, immunoconjugates, small molecule antagonists, immunotherapy, RNAi-mediated EphA2 silencing, EphA2-derived peptides and targeting EphA2 using nanoparticles. Combination of EphA2-directed therapies with other anticancer therapies has also been evaluated [6, 7].

High expression of EphA3 has also been observed in many types of solid cancers and in leukaemias [4]. A monoclonal antibody targeting EphA3 has been proven to be successful in pre-clinical studies and has been shown to have no significant toxicity in clinical studies [4, 8]. Anti-EphA3 antibody therapy has been shown to have a tumour-specific targeting role and, in some tumours, an effect on the tumour microenvironment [2, 5]. Anti-EphA3 monoclonal antibody therapy can target through antibody- dependent cell-mediated cytotoxicity (ADCC) and can also be used to target toxic “payloads” to the tumour. The EphA3 monoclonal antibody induces receptor endocytosis, thus can effectively deliver radioactive isotopes, including the alpha particle emitting isotope bismuth (213Bi), and cytotoxic drugs specifically into the tumour cell. These agents, acting in concert with direct functional effects of the monoclonal antibody, have shown a greatly enhanced anti-tumour effect compared with un-modified antibody [2, 3, 5].

EphB4 expression has been detected in both embryonic vasculature and in tumour angiogenesis and progression; it plays an important role in cell signalling, morphology, adhesion, migration and invasion. This includes EphB4 signalling which leads to activation of downstream signals including VEGF expression. EphB4 also has a tumour suppressor effect in some cancers including colon and breast. The differing roles of EphB4 in tumour progression have led to the development of many strategies in therapeutic targeting of this protein. One of these approaches is the use of a therapeutic antibody which results in EphB4 degradation, thereby inhibiting tumour angiogenesis and tumour growth. Another approach has been to competitively block EphB4-ephrinB2 signalling using a soluble, monomeric-human serum albumin (EphB4-HSA) fusion protein as a decoy receptor. In pre-clinical models, this approach has been successfully exploited to inhibit tumour growth in colon, lung, breast, glioma, melanoma and prostate cancer [2, 4].

In summary, we and others have developed agents which target Eph proteins as novel therapeutics which have been tested successfully in pre-clinical and early phase clinical studies. Because the Eph RTKs are expressed at low levels in normal adult tissues, several therapies targeting members of the Eph RTK family have been shown to have a highly favourable toxicity profile making them relatively tumour- specific agents. As we move into an era of individually tailored therapies based on tumour phenotype, the development of anti-Eph therapies will provide an important, low toxicity addition to the therapeutic arsenal.
